# The *Arabidopsis* alkaline ceramidase TOD1 is a key turgor pressure regulator in plant cells

**DOI:** 10.1038/ncomms7030

**Published:** 2015-01-16

**Authors:** Li-Yu Chen, Dong-Qiao Shi, Wen-Juan Zhang, Zuo-Shun Tang, Jie Liu, Wei-Cai Yang

**Affiliations:** 1 State Key Laboratory of Molecular Developmental Biology and National Center for Plant Gene Research (Beijing), Institute of Genetics and Developmental Biology, Chinese Academy of Sciences, Beijing 100101, China; 2 University of Chinese Academy of Sciences, Beijing 100049, China; 3 Collaborative Innovation Center for Genetics and Development, Fudan University, Shanghai 200433, China

## Abstract

Turgor pressure plays pivotal roles in the growth and movement of walled cells that make up plants and fungi. However, the molecular mechanisms regulating turgor pressure and the coordination between turgor pressure and cell wall remodelling for cell growth remain poorly understood. Here, we report the characterization of *Arabidopsis TurgOr regulation Defect 1* (*TOD1*), which is preferentially expressed in pollen tubes and silique guard cells. We demonstrate that TOD1 is a Golgi-localized alkaline ceramidase. *tod1* mutant pollen tubes have higher turgor than wild type and show growth retardation both in pistils and in agarose medium. In addition, *tod1* guard cells are insensitive to abscisic acid (ABA)-induced stomatal closure, whereas sphingosine-1-phosphate, a putative downstream component of ABA signalling and product of alkaline ceramidases, promotes closure in both wild type and *tod1*. Our data suggest that TOD1 acts in turgor pressure regulation in both guard cells and pollen tubes.

## Abstract (web summary)

Turgor pressure is critical for the growth of plant cells but the mechanisms regulating turgor are poorly understood. Here, Chen *et al*. identify TOD1, an alkaline ceramidase, involved in sphingosine metabolism that regulates turgor during pollen tube growth and stomatal closure.

## Graphical abstract

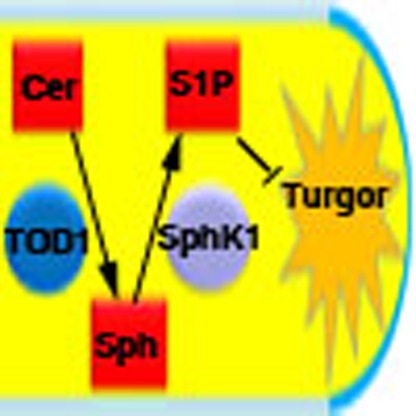

A fundamental question in biology is what drives cells to grow and migrate. In animal cells, the cytoskeleton acts as the driving force^1^,^2^, whereas in the walled cells that make up plants and fungi, turgor pressure is thought to play pivotal roles^3^,^4^,^5^. Cellular turgor pressure is exerted by water moving in response to cellular concentration of ions, sugars and other solutes. Turgor pressure is necessary for cell enlargement, growth and shape maintenance. Pollen tubes and guard cells are two types of highly specialized plant cells, which evolved to allow adaption to life on land^6^,^7^. In guard cells, turgor pressure is the driving force for stomatal movement. In pollen tubes, turgor pressure is also essential for tip growth. Although turgor pressure is not directly correlated with growth rate, tube growth stops when the turgor pressure is lower than a threshold level^8^,^9^. Before reaching a plateau, the pollen tube growth rate increases as turgor pressure rises^10^. Furthermore, increased external pressure reduces the growth rate^8^,^10^. Although these *in vitro* studies demonstrated that turgor pressure is critical for pollen tube growth, the mechanisms of turgor pressure regulation and the coordination between turgor pressure and the cell wall for *in vivo* growth, that have attracted plant biologists for a long time are still poorly understood.

In angiosperms, after landing on the stigma, pollen grains hydrate and germinate, and each produces a pollen tube—a single-celled structure that delivers the sperm cells to the ovule^11^. A pollen tube penetrates into the style, and into the transmitting tract, then emerges onto the surface of the septum and grows along the funiculus and is targeted to the micropyle of the ovule^12^,^13^. The pollen tube finally bursts when it reaches one of the two synergid cells of the ovule, and the two sperm cells are released to enable double fertilization^14^. During this process, pollen tubes need to pass through different tissues of the pistil including papillar cells of the stigma, the transmitting tract, the septum and the filiform apparatus^13^. To penetrate these ‘physical barriers’ of the pistil, pollen tubes must regulate their turgor pressure during *in vivo* growth.

Sphingolipids are ubiquitously present in all eukaryotic cells and in a few bacteria^15^. They are thought to be essential for membrane structure and are also involved in signal transduction, controlling essential cellular processes such as apoptosis, cell migration, differentiation and inflammation^15^. Ceramide, sphingosine, sphingosine-1-phosphate (S1P) and their derivatives are examples of a class of well-studied bioactive sphingolipids in animals. Ceramidases hydrolyse ceramide to yield sphingosine and fatty acid. They play a key role in sphingolipid metabolism, being responsible for regulatory activity at the cellular level. Ceramidases are classified as acidic, neutral or alkaline, based on their optimal pH for biological activity. Based on sequence homology, the *Arabidopsis* genome encodes one alkaline phytoceramidase^16^ and three neutral ceramidases^17^, but their functions have not been characterized.

Here, we show that TurgOr regulation Defect 1 (TOD1) is a *bona fide* alkaline ceramidase that is preferentially expressed in pollen tubes and in silique guard cells, where it is required for turgor pressure regulation. Disruption of *TOD1* results in partial male sterility, which is due to defects of pollen tube growth in penetrating the pistils. *tod1* mutant pollen tubes also show growth retardation inside agarose medium; this retardation is rescued by application of sphingosine or S1P. These data suggest that maintenance of turgor pressure likely controlled by TOD1 is needed for pollen tube growth in pistil. Furthermore, our work provides novel insights into the role of sphingolipid signalling pathways during pollen tube growth in plants.

## Results

### Isolation of the *tod1* mutant

**Figure 1:**
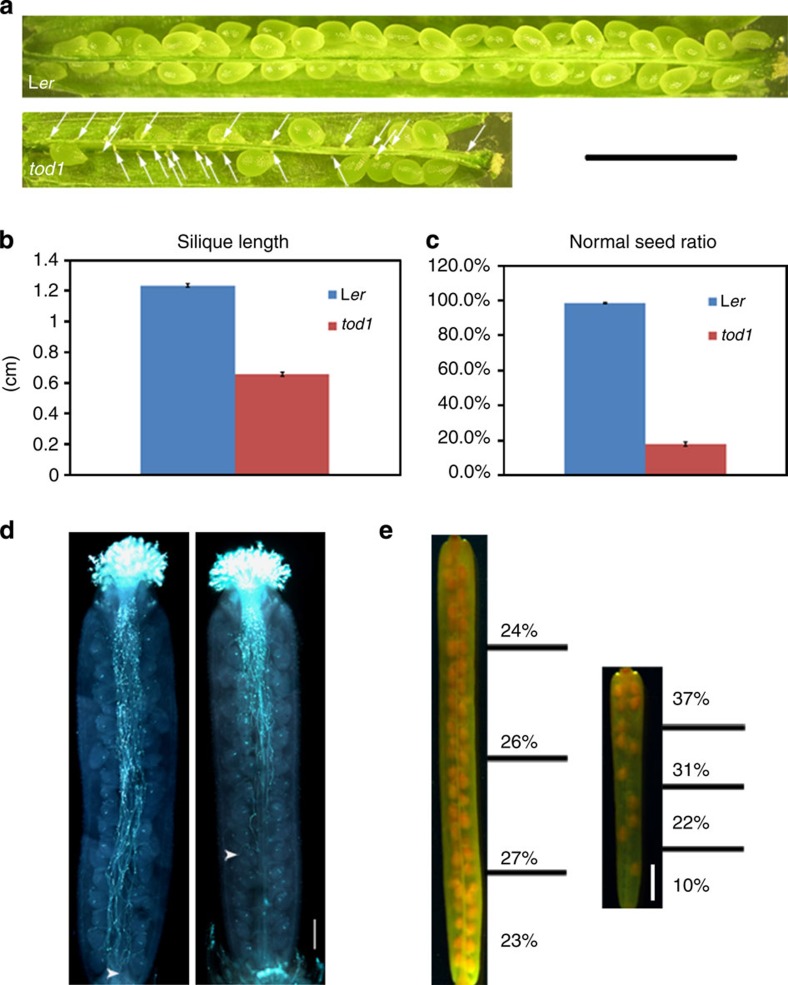
Phenotype of *tod1* mutant. (**a**) Dissected siliques from L*er* and *tod1*. Arrows point to aborted ovules. Scale bar, 2 mm. (**b**) Silique lengths of L*er* and *tod1*. Data are mean±s.e. (*n*=72). (**c**) Seed ratios of L*er* and *tod1*. Data are mean±s.e. (*n*=33). (**d**) *tod1* pollen tubes showed growth defects *in vivo*. L*er* pollen tubes (left panel) and *tod1* pollen tubes (right panel) in L*er* pistils stained with aniline blue 24 h after pollination. The longest pollen tubes are indicated by arrowheads. Scale bar, 200 μm. (**e**) Seed distribution in sliques from L*er* (left panel) and *tod1* (right panel) selfed plants. Siliques were cleared by ethanol and images were recorded using a stereo microscope equipped with CCD (L*er*, *n*=42; *tod1*, *n*=43). Scale bar, 1 mm.

We screened gene trap lines of *Arabidopsis thaliana* ecotype Landsberg *erecta*^18^ for mutants defective in pollen tube growth *in vivo* and thereby identified one mutant, designated *TurgOr regulation Defect 1* (*tod1*). Mutant plants bore short siliques resulting from severe sterility (Fig. 1a). The average silique length of homozygous *tod1* was about one-half that of the wild-type (Fig. 1b) and only about 18% of the available ovules were fertilized to produce seeds (Fig. 1c). The F_1_ progeny of the heterozygous mutant showed a 1:1 (271:265, *χ*^2^<*χ*^2^_0.01_) segregation ratio of kanamycin-resistant (Kan^R^) to kanamycin-sensitive (Kan^S^), indicating that the *Ds* insertion affect the function of either the male or female gametophyte^19^. To determine which sex was affected, we conducted reciprocal crosses. When wild type was used as a pollen donor on heterozygous *tod1* flower, the segregation ratio of the F_1_ progeny was 0.94:1 (191:203), indicating that the kanamycin-resistant gene *NPTII* was transmitted by the female gametophyte. However, when pollen from heterozygous *tod1* was used to pollinate wild-type flowers, the segregation ratio of Kan^R^:Kan^S^ in the F_1_ progeny was 0.025:1 (15:601), indicating that *NPTII* was barely be transmitted by the male gametophyte. These data confirmed that the mutation affects the male gametophyte.

### *tod1* is defective in *in vivo* pollen tube growth

To investigate the cause of male sterility, mature pollen grains were stained with Alexander’s stain and with 4′,6-diamidino-2-phenylindole (DAPI) and analysed by microscopy. The cytoplasm of *tod1* pollen was red purple after Alexander’s staining (Supplementary Fig. 1a), indicating that the viability of the mutant pollen grains was not affected^20^. DAPI staining showed two bright sperm nuclei and one more diffuse vegetative nucleus, as found in wild-type plants (Supplementary Fig. 1b). To determine whether the mutation caused defects in pollen germination and/or pollen tube growth, we checked *in vitro* pollen germination rates and tube growth. Both wild-type and the mutant pollen yielded above 75% germination after 5 h incubation (Supplementary Fig. 1c). There were also no differences between *in vitro* pollen tube length of wild type and mutant after 5 h germination (Supplementary Fig. 1d).

To further check whether *tod1* had defects in *in vivo* pollen tube growth, wild-type and mutant pollen tubes growing in L*er* pistils were stained with aniline blue. *tod1* pollen tubes showed reduced growth potential (Supplementary Fig. 2), and only a few pollen tubes had reached the middle of the pistil 24 h after pollination, whereas wild-type pollen tubes had reached the base of the pistil (Fig. 1d and Supplementary Fig. 2). We also checked the status of pollen tubes 48 h after pollination, when almost all ovules were fertilized and pollen tubes began to degenerate in the wild type (Supplementary Fig. 3); most *tod1* pollen tubes were still in the top half of the pistil, and many of them had begun to degenerate (Supplementary Fig. 3). Consistent with the *in vivo* phenotype of pollen tubes, the seeds distribution in *tod1* siliques was distorted, with more seeds at the stigma end (Fig. 1e). It seemed that the mutant pollen tubes had lost the ability to penetrate efficiently through the style.

To confirm whether the low seed set phenotype was also caused by defects in pollen tube guidance in the gametophytic phase, we observed *tod1* pollen tubes in detail by aniline blue staining and scanning electron microscopy (SEM). Once the mutant pollen tubes penetrated the septum walls, they grew along the funiculus and then entered the micropyle of the ovule to complete fertilization as seen for wild-type tubes (Supplementary Fig. 4a–d). Limited pollinations showed that only about 10% of *tod1* pollen tubes in the transmitting tract could go through the septum wall; more than 95% of septum-penetrating *tod1* pollen tubes could enter the micropyle of the ovule (Supplementary Fig. 4e). This suggests that pollen tube guidance in the gametophytic phase was not affected in the mutant.

### Molecular cloning of *TOD1*

**Figure 2:**
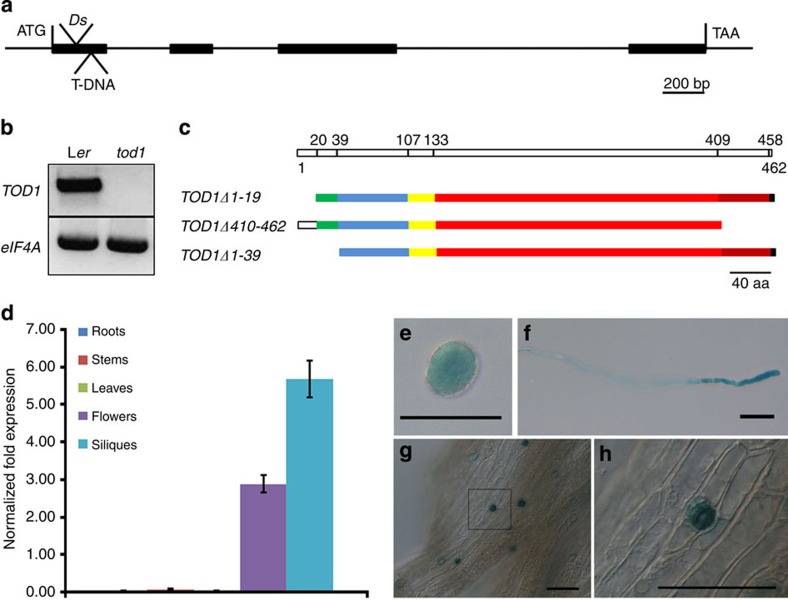
Detection of mutations causing partial male sterility in *tod1* mutants and expression pattern of *TOD1*. (**a**) Genomic structure of *TOD1*. *Ds* and T-DNA inserted into the first exon at 114-bp and 186-bp downstream of start codon in *tod1-1* and *tod1-2*, respectively. (**b**) Full-length *TOD1* transcripts are absent in the mutant. Total RNA from inflorescences was reverse-transcribed and amplified by PCR. (**c**) Scheme of TOD1. It contains a transmembrane domain at the N terminus (residues 20–39), and a DUF616 domain (residues 107–409). Amino acids 133–458 are annotated as an alkaline phytoceramidase domain by PANTHER. (**d**) Real-time RT–PCR of *TOD1* expression pattern in different tissues of *Arabidopsis*. Data are mean±s.e. (*n*=4). (**e**–**h**) Cell-specific *pTOD1::gTOD1*-*GUS* expression. (**e**) Mature pollen grain, (**f**) pollen tube, (**g**) guard cells on a silique and (**h**) magnification of the area indicated in **g**. Scale bars, 50 μm.

We performed thermal asymmetric interlaced PCR^21^ to obtain the flanking genomic sequence of the *Ds* element. Sequencing indicated that the *Ds* element was inserted into the first exon of *At5g46220*, 114-bp downstream of the ATG (Fig. 2a). The *Ds* insertion resulted in an 8-bp duplication (5′-CTCTCAAG-3′) at the insertion site. Furthermore, we were unable to detect *TOD1* transcripts in inflorescence RNA, indicating that *tod1* was a null allele (Fig. 2b).

To confirm that the *Ds* insertion in *TOD1* was indeed the cause of the mutant phenotype, we transformed a 5,557-bp genomic DNA fragment of *TOD1*, containing the predicted promoter region, open reading frame and 3′-untranscribed region (3′-UTR) into homozygous *tod1* plants. Eighty-seven independent T1 transgenic lines were obtained. We randomly selected ten lines for analysis of silique length and seed set. Fertility was restored in complemented mutant plants (Supplementary Table 1), indicating that *TOD1* was sufficient to rescue pollen tube growth defect. In addition, SALK_110396, which harboured a T-DNA insertion in the first exon of *At5g46220* (Fig. 2a), named *tod1-2*, also displayed the similar phenotype as observed in *tod1-1*.

*TOD1* is predicted to encode a 462-amino-acid protein, with a transmembrane domain (residues 20–39), and a DUF616 domain (residues 107–409; Fig. 2c), but no other homology to proteins with known functions. However, the PANTHER (Protein ANalysis THrough Evolutionary Relationships) analysis classified TOD1 as a member of a family of alkaline ceramidases, although they have no similarity at the amino-acid level (http://www.pantherdb.org/)^22^,^23^. We generated a phylogenetic tree using TOD1 orthologues from other plant species and other eukaryotic organisms, all of which belong to the alkaline ceramidase family. Interestingly, these members clustered into two clades: one contains plant-specific proteins and the other includes proteins belonging to other eukaryotic organisms (Supplementary Fig. 5a). The plant-specific clade, including TOD1 from *Arabidopsis*, contains members from eudicots including grape (*Vitis vinifera*), soybean (*Glycine max*), *Medicago truncatula*, poplar (*Populus trichocarpa*) and monocots such as rice (*Oryza sativa*). These members’ functions are currently not known. The other clade contains members from yeast (*Saccharomyces cerevisiae*), human (*Homo sapiens*) and mouse (*Mus musculus*). Their functions have been characterized as alkaline ceramidases^24^,^25^,^26^, alkaline phytoceramidases^16^,^27^ or alkaline dihydroceramidases^28^,^29^. The sequence similarity between these two clades is very low, but they share high similarity within their clades (Supplementary Fig. 5b).

The region comprising amino acids 133–458 is predicted as a putative alkaline phytoceramidase domain (Fig. 2c). To test the function of the gene, we constructed three deletion variants of *TOD1* to complement the mutant phenotype (Fig. 2c). Only *TOD1Δ1-19*, which contains the transmembrane domain and the putative alkaline phytoceramidase domain, could restore the seed set of the mutant. Neither *TOD1Δ410-462* nor *TOD1Δ1-39* could complement the mutant phenotype. These results indicate that both the transmembrane domain and putative phytoceramidase domain are critical for TOD1 function.

### *TOD1* is expressed in pollen and silique guard cells

**Figure 3:**
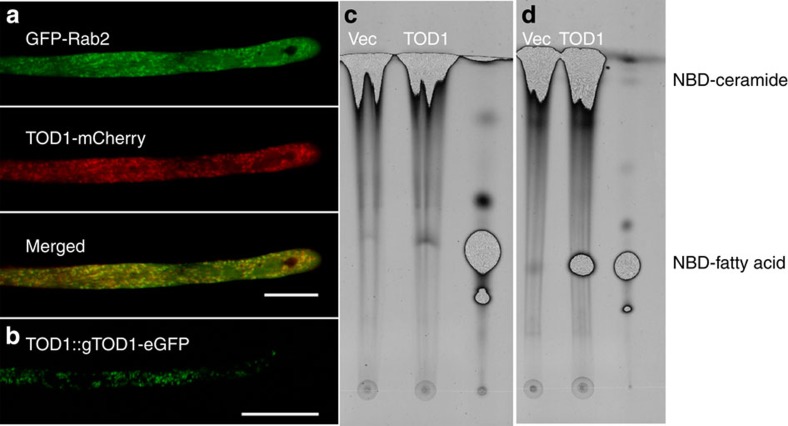
TOD1 is a *bona fide* alkaline ceramidase localized in the Golgi apparatus. (**a**) CLSM images showing that TOD1-mCherry co-localizes with Golgi marker GFP-Rab2. *pLat52::TOD1-mCherry* and *pLat52::GFP-Rab2* were co-transfected and transiently expressed in tobacco pollen tubes. Scale bar, 20 μm. (**b**) TOD1-eGFP localization in an *Arabidopsis* pollen tube from plants stably transformed with *pTOD1::gTOD1-eGFP*. Scale bar, 20 μm. (**c**,**d**) TOD1 has alkaline ceramidase activity. Expression of TOD1 was induced in yeast mutant Δ*ypc1*Δ*ydc1*, which lacks two endogenous yeast ceramidases. Total microsomes from Δ*ypc1*Δ*ydc1* containing empty vector pYes2 (Vec) or pYes2-Flag-TOD1 (TOD1) incubated with the fluorescent substrate NBD-ceramide. The third lane is the NBD-fatty acid standard. (**c**) pH 7.0. (**d**) pH 9.4.

Real-time reverse transcription (RT)–PCR showed that *TOD1* transcripts were present in flowers and siliques, were barely detectable in roots, stems or leaves (Fig. 2d). To further investigate the cellular expression pattern of *TOD1*, we examined *pTOD1::gTOD1*-*GUS* transgenic lines which express β-glucuronidase (GUS); *TOD1* was preferentially expressed in pollen grains, pollen tubes and silique guard cells (Fig. 2e-h). To characterize the subcellular localization of TOD1, we co-transformed *pLat52::TOD1-mCherry* with endomembrane system markers into tobacco (*Nicotiana tabacum*) pollen grains by bombardment. Confocal laser scanning microscopy (CLSM) showed that TOD1 co-localized only with Golgi marker GFP-Rab2 (Fig. 3a). Moreover, pollen tubes from transgenic *Arabidopsis* plants expressing TOD1-enhanced green fluorescent protein (eGFP) under its native promoter also showed similar localization pattern (Fig. 3b). Thus, TOD1 is a Golgi-localized protein in pollen tubes.

### TOD1 is a *bona fide* alkaline ceramidase

To test whether TOD1 has ceramidase activity, we inserted the full-length *TOD1*-coding sequence with a FLAG tag at its amino terminus into the yeast expression vector pYES2, under the control of the *GAL1* promoter. The resulting construct was transformed into the yeast mutant Δ*ypc1*Δ*ydc1*, lacking two endogenous yeast ceramidases YPC1p and YDC1p^28^. The expression of Flag-tagged TOD1 was induced by galactose and confirmed by western blot analysis using an anti-Flag antibody (Supplementary Fig. 6a). Total microsomal extracts were prepared from yeast cells and used for enzyme activity assays with NBD-C_12_-phytoceramide (with the C-4 hydroxyl group) or NBD-C_12_-ceramide (with the Δ4-double bond) as substrate. The thin-layer chromatography (TLC) showed that phytoceramide is not a substrate for TOD1 (Supplementary Fig. 6b), but ceramide is (Fig 3c,d). Furthermore, TOD1 displayed stronger ceramidase activity at pH 9.5 than at pH 7.0 (Fig. 3c,d). Taken together, we conclude that TOD1 is an alkaline ceramidase.

### TOD1 regulates turgor in guard cells and pollen tubes

**Figure 4:**
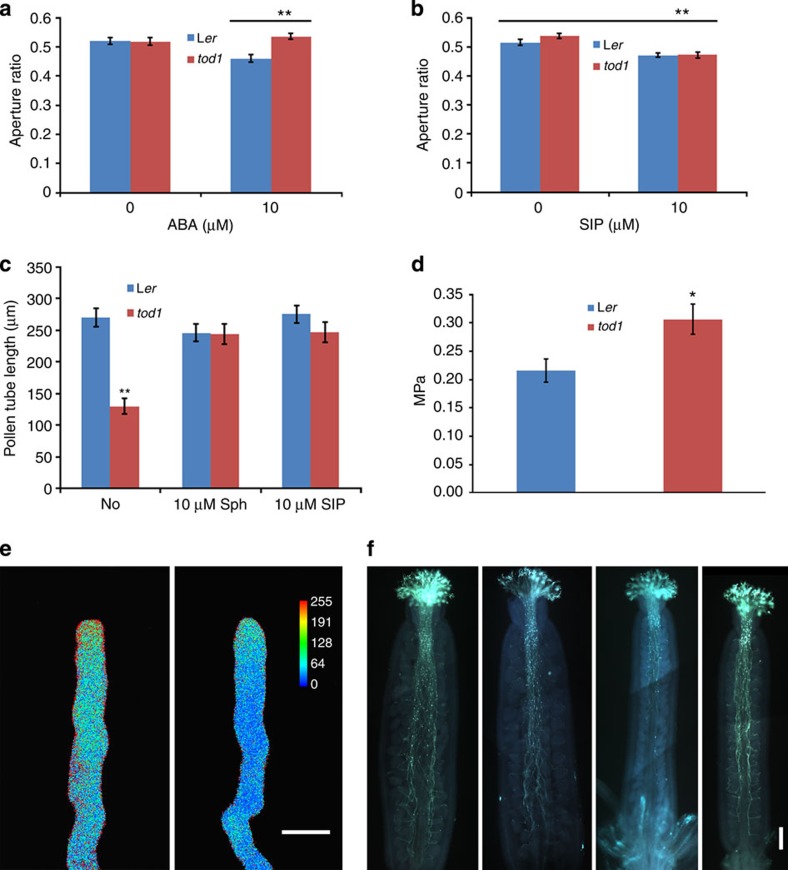
*tod1* mutants have a turgor problem. (**a**) Exogenous 10 μM ABA promotes pre-opened stomata closure in L*er*, but not in *tod1.* Data are mean (width/length)±s.e. (*n*=48). (**b**) Exogenous 10 μM S1P promotes closure of pre-opened stomata both in L*er* and *tod1.* Data are mean (width/length)±s.e. (*n*=48). (**c**) *tod1* pollen tubes grow slower than those of wild type inside pollen tube germination medium with 1.5% agarose. Growth defects of mutant pollen tubes can be rescued by adding 10 μM sphingosine (Sph) or S1P. Data are mean±s.e. (*n*>55). (**d**) *tod1* pollen tubes generate a higher turgor pressure than wild-type pollen tubes. Data are mean±s.e. (L*er*, *n*=19; *tod1*, *n*=10). (**e**) Typical YC3.60 cameleon Venus/ECFP ratio images of maximum [Ca^2+^]_cyt_ in growing pollen tubes. Left panel, Col pollen tube expressing YC3.60. Right panel, *tod1-2* pollen tube expressing YC3.60. The coloured bar represents the relative cytosolic Ca^2+^ levels. Scale bar, 10 μm. (**f**) Suppression of *tod1-2* pollen tube growth defect by *gaut13-1* mutation. Col, *gaut13-1*, *tod1-2* and *tod1-2 gaut13-1* double mutant pollen tubes in Col pistils (from left to right) stained with aniline blue 12 h after pollination. Scale bar, 200 μm. Student’s *t*-test, *P<0.05; **P<0.01.

As *TOD1* is expressed both in pollen tubes and in silique guard cells, we checked whether the stomata on siliques had defects in response to abscisic acid (ABA) or S1P. Stomata on siliques had similar response to ABA as stomata on leaves (Fig. 4a). ABA at concentration of 10 μM promotes pre-opened stomata closing in L*er*, but not in *tod1* (Fig. 4a), indicating that *tod1* is insensitive to exogenous ABA. ABA can activate sphingosine kinases (SphKs), which will phosphorylate sphingosine, the product of the ceramidase reaction, and subsequently increase S1P level^30^. S1P is known to play a positive role in stomatal closure^31^. Interestingly, exogenous S1P (10 μM) promoted closure of pre-opened stomata both in L*er* and *tod1* (Fig. 4b). These data suggested that TOD1 is also involved in ABA-induced guard cell turgor regulation, likely acting downstream of ABA and upstream of S1P.

To confirm whether the *in vivo* growth defects of *tod1* pollen tubes could be caused by altered turgor pressure, we germinated pollen grains inside germination medium supplemented with 1.5% agarose. After 10 h growth, the average pollen tube length of *tod1* pollen was about 130 μm, half of the wild-type length (Fig. 4c). As there was no difference in pollen tube growth between wild-type and *tod1* on agarose medium as shown earlier (Supplementary Fig. 1d), it seemed possible that the mutant pollen tubes were defective in turgor pressure regulation, thus leading to the reduced penetration potential. To further check whether sphingosine or S1P could rescue the growth defects of *tod1* pollen tubes inside the germination medium with 1.5% agarose, we measured the average length of mutant pollen tubes germinated in the presence of 10 μM sphingosine or S1P. The average length of mutant pollen tubes increased from 130 μm (without) to about 245 μm (with sphingosine or S1P; Fig. 4c), indicating that both sphingosine and S1P can rescue the growth defects of mutant pollen tubes with higher external pressure.

To test whether the *tod1* mutation affected turgor pressure, we tried to measure pollen tube turgor pressure by the pressure probe and incipient plasmolysis methods. However, *Arabidopsis* pollen tubes are too small for the pressure probe, so instead we used the incipient plasmolysis method for turgor pressure measurement. The *tod1* pollen tubes generated higher turgor pressure than wild-type pollen tubes (Fig. 4d). Therefore, we conclude that a mutation in the *TOD1* gene results in disregulation of turgor pressure.

As S1P is involved in the regulation of [Ca^2+^]_cyt_ in animal cells and guard cells, we also examined [Ca^2+^]_cyt_ in growing pollen tubes with a YC3.60 cameleon probe. The Venus/ECFP ratio imaging showed that the *tod1* pollen tubes had decreased [Ca^2+^]_cyt_ (Fig. 4e).

To provide further genetic evidence for TOD1’s role in turgor regulation, we screened for extragenic suppressors of the *tod1* mutation and isolated one suppressor that rescued the reduced seed set defect of *tod1*. Whole-genome re-sequencing and SNP (single-nucleotide polymorphism) analysis results revealed that the suppressor mutant had a single-nucleotide substitution in the *Galacturonosyltransferase13* (*GAUT13*) gene, resulting in a premature stop codon. GAUT13 and its homologue GAUT14 function redundantly in pollen tube growth, possibly through pectin biosynthesis of the pollen tube wall^32^. Pollen tubes of the *gaut13 gaut14* double mutant are swollen and defective in elongation, both in *in vitro* and *in vivo*^32^. To confirm the re-sequencing result, we crossed two T-DNA insertion lines of *tod1-2* and *gaut13-1*, and obtained homozygous double mutants in the F_2_ generation. The pollen tube growth defects of *tod1-2* were indeed suppressed by the *gaut13* mutation (Fig. 4f). Double mutant pollen tubes arrived at the base of the pistil 12 h after pollination, and grew through the septum wall and target the micropyle of the ovule (Fig. 4f). The seed ratio of double mutant plants was restored from the 23% seen with the *tod1-2* single mutant to 89% (Supplementary Fig. 7).

## Discussion

To date, the role of turgor pressure in pollen tube growth is controversial^33^,^34^,^35^,^36^. Here, we identified a *Ds* insertion mutant, *tod1*, in which *in vivo* pollen tube growth was defective. Disruption of *TOD1* caused partial male sterility, resulting from the reduced growth potential of mutant pollen tubes. Detailed examination indicated that the ability of *tod1* pollen tubes to penetrate pistil tissues was severely compromised. *tod1* pollen tubes also showed growth defects inside medium solidified with 1.5% agarose, in which the external pressure was higher than normal conditions. We attributed this inability to disregulation of turgor pressure of the mutant pollen tubes.

Pollen tube growth is regulated by the coordination of turgor pressure and cell wall stiffness. Turgor pressure is thought to be the driving force, whereas the cell wall is thought to be the speed setter. For example, application of high concentrations of pectinase caused apical swelling or bursting of pollen tubes, whereas application of moderate concentration pectinase stimulated tube growth^37^. It was therefore hypothesized that pectinase that digest pectins, the main cell wall component of pollen tubes, sets the tube growth rate by regulating the physical characteristics of the tube cell wall. Silencing of the gene encoding tobacco pollen pectin methylesterase involved in the building up of the pollen tube wall strength, resulted in retarded *in vivo* pollen tube growth, whereas *in vitro* pollen tube growth was not affected^38^. This suggests that the stiffness of pollen tube wall should be kept at an optimum. Turgor pressure also needs to be maintained at an optimal and in a narrow range, neither too high nor too low, although the growth rate is likely not correlated with turgor pressure^8^. *tod1* pollen tubes generate higher turgor pressure than the wild-type ones as measured by the incipient plasmolysis method (Fig. 4d). Much higher turgor pressure is expected during *in vivo* growth since pollen tubes need to resist external pressure from pistil tissues during their penetration. Why are mutant tubes with higher turgor pressure defective in *in vivo* growth? One explanation is that the turgor pressure of the mutant tubes, exceeding the *in vivo* pressure optimal for wild-type tubes, impairs tube growth. In addition, the increased turgor pressure in *tod1* pollen tubes may also affect the building up of the pollen tube wall strength, which might also cause *in vivo* retarded growth. To resist the increased turgor pressure from bursting, *tod1* mutant pollen tubes might deposit much more wall material, which in turn would display reduced growth potential. Finding that *gaut13* acts as a suppressor of *tod1* supports this hypothesis, because mutation of *GAUT13* has quantitative effects on cell wall deposition.

Sphingolipids are ubiquitous in eukaryotes and some bacteria. The biosynthesis and metabolism of sphingolipids have been well studied for the last two decades. Ceramide is mainly synthesized in the ER (endoplasmic reticulum), and is then transported to the Golgi apparatus for further modifications. *TOD1* shares low sequence similarity with reported alkaline ceramidases from human^25^ and mouse^24^. We showed here that TOD1 possesses ceramidase activity optimally at alkaline pH and it is localized to the Golgi apparatus. Human alkaline ceramidase 2 (haCER2), which shows a pH optimum of 8.0, is also localized to the Golgi apparatus^25^. In eukaryotic cells, different organelles exhibit variable pH to fulfill different requirements for protein function within them. Although the average pH value of the Golgi is about 6.4 (ref. 39), it can be alkalinized by Na^+^/H^+^ exchangers on its membrane^40^. It is possible that rapid activation of these ion exchangers might generate highly alkalinized area in close proximity to Golgi membranes. Furthermore, TOD1 also had weak ceramidase activity at pH 7.0, so it is likely that a basal level of activity is present in the Golgi at neutral pH. The possible coupling between TOD1 activity and the activation of ion exchangers in Golgi membrane is of special interest for further investigation.

In animal cells, ceramide, sphingosine, S1P and their derivatives are attracting considerable interest because of their roles in intra- and extracellular signalling. However, recent studies using lipidomic analyses suggested that the abundance of Δ4-unsaturated sphingolipdids is low in *Arabidopsis*^41^,^42^. This is not surprising that, because work from Michaelson and colleagues^42^ and our data, several key enzymes involved in metabolism of Δ4-unsaturated sphingolipdids metabolism are restrictedly expressed in limited cell types. Therefore, sphingolipds signalling may function only in specific plant cells. S1P had been proposed to act positively in stomatal closure from pharmacological experiments^30^. Stomatal activity is also regulated by turgor pressure. Our results indicate that stomatal closure is defective in guard cells of *tod1* siliques, and this can be rescued by exogenous application of S1P, confirming that TOD1 acts upstream of S1P in stomatal closure. In pollen tubes, disruption of *TOD1* also results in higher turgor pressure, and the pollen tube growth retardation caused by increased external pressure can be rescued by exogenous application of S1P. These findings suggest that TOD1 acts similarly both in guard cells and pollen tubes, where it is preferentially expressed.

Based on sequence homology, six *SphKs* were identified in the *Arabidopsis* genome^43^,^44^, three of which *LCBK1* (ref. 45), *SphK1* (ref. 43) and *SphK2* (ref. 44), were confirmed to have kinase activity towards sphingosine. As these three *SphK* genes are all expressed in pollen tubes^46^, and *SphK1* and *2* are located in the same locus^44^, we generated an artificial miRNA-based knockdown allele targeting *SphK1* and *2* in *lcbk1* background. However, these transgenic lines did not show *tod1*-like pollen tube phenotype in pistil. This is most likely due to genetic redundancy, and there may be other genes, acting as SphK, which have not been characterized yet.

In conclusion, we demonstrated that TOD1 is a *bona fide* alkaline ceramidase that plays a critical role in turgor pressure regulation during pollen tube growth *in vivo* and in stomatal closure. This opens up an opportunity to dissect the role of sphingolipids in turgor pressure control in plant cells. Further studies on S1P signalling and sphingolipidomics will likely provide novel insights into turgor pressure regulation in plant cells.

## Methods

### Plant materials and growth conditions

*Arabidopsis thaliana* ecotypes Landsberg *erecta* (L*er*) or Columbia (Col) were used as the wild type. *tod1-2* (SALK_110396) was obtained from Arabidopsis Biological Resource Center (ABRC)^47^. *gaut13-1* (SALK_150132) was obtained from Li-Qun Chen (China Agricultural University)^32^. YC3.60 transgenic seeds were obtained from Megumi Iwano (Nara Institute of Science and Technology)^48^. Surface sterilized seeds were germinated on Murashige and Skoog (MS) medium supplemented as required with 50 mg l^−1^ kanamycin and/or 20 mg l^−1^ hygromycin. The seeds were kept at 4 °C for 3 days, and then transferred to a greenhouse (22 °C) under long-day conditions (16 h light/day). Seedlings were transplanted to soil 10 days after plating.

### Mutagenesis and screens for suppressors of *tod1*

Approximately 5,000 *tod1* seeds were mutagenized by imbibition of 0.3% ethyl methanesulfonate for 12 h at room temperature, followed by washing once with 100 mM Na_2_S_2_O_3_ and several times with sterilized water. The M_2_ seeds were collected in 32 pools, each representing approximately 280 M_1_ plants. We screened approximately 18,000 M_2_ seeds for suppressors of *tod1*. Putative suppressors were backcrossed to *tod1* before further genetic and phenotypic analyses. Plants homozygous for the *tod1* allele were confirmed by PCR analysis. BC_4_F_2_ of putative suppressors were crossed to *tod1-2*, and the resulting F_2_ populations were examined for the segregation of fertility. Dominance or recessiveness of putative suppressors was determined by segregation ratio of full seed set to reduced seed set. For recessive mutations, genomic DNA was isolated from individual F_2_ plants with full seed set. For dominant mutations, seeds from individual F_2_ plants with full seed set were collected and propagated separately. When all individual F_3_ plants from the same F_2_ showed restored seed set, then genomic DNA was isolated from individual F_3_ plants.

### Whole-genome re-sequencing and data analysis

DNA was isolated using a DNeasy Plant Mini Kit (Qiagen). Equally pooled genomic DNA was used to prepare a library for Illumina sequencing. The library was sequenced on the HiSeq2000 platform and 100 bp paired-end reads were generated. Reads alignment and variants identification were done according to the references with modifications^49^,^50^. Clean reads were mapped against the TAIR10 releases of the *Arabidopsis* genome, using the BWA software with default parameters^51^. Sites with more than two alleles were not considered. To eliminate bias from cross breeding, variants were removed that existed in the variant list (http://1001genomes.org/data/MPI/MPISchneeberger2011/releases/2012_03_14/Ler-1/Marker/Ler-1.SNPs.TAIR9.txt). SNPs were further filtered with two steps: (i) extraction of SNPs that exhibit G/C-to-A/T transitions, which are the most frequent changes caused by ethyl methanesulfonate mutagenesis. (ii) SNPs with a supporting reads depth less than 20 or more than 100 were removed to reduce mapping errors. Gene mapping analysis was performed according to the reference^51^ with default parameters except for SNPs filtering, which indices more than 0.2 were considered. The SNP locations within the gene model were annotated using SNPEff V3.3. Genes with ‘HIGH’ impact SNPs were taken as candidates.

### Pollen and pollen tube staining

Mature pollen grains were stained for DNA with 1 μg ml^−1^ DAPI for 10 min (ref. 52), and for pollen viability with Alexander’s stain^53^. For pollen tube staining, the pistil carpel was partially dissected using a forceps and a syringe needle, and then was fixed in acetic acid/ethanol (1:9) solution for more than 2 h. The fixed tissue was softened in 1 M NaOH overnight, and then stained with 0.01% aniline blue in 50 mM potassium phosphate buffer (pH 7.5). The stained samples were then observed with a Zeiss Axioplan microscope (Carl Zeiss) equipped with epifluorescence.

### *In vitro* pollen germination and pollen tube growth

Pollen grains from fully opened flowers were spread on pollen germination medium containing 5 mM CaCl_2_, 5 mM KCl, 1 mM MgSO_4_, 0.01% H_3_BO_3_, 10% sucrose (w/v) and 1.5% agarose (for solid medium), pH 7.5, and incubated at 22 °C (ref. 54). The germination rate and pollen tube lengths were determined with a Zeiss Axioplan microscope.

Sphingosine and S1P were dissolved in methanol to make stock solution. The complementation experiment were conducted in 1.5% ultra-low gelling agarose (Sigma-Aldrich) germination medium with additions of 10 μM Sphingosine (860490 P, Avanti) or S1P (S9666, Sigma), or the same volume of methanol for the control.

Liquid pollen tube germination was performed in 2 ml Eppendorf tube, using 20 flowers for each 200 μl medium.

### Scanning electron microscopy

Pistils pollinated with either L*er* or *tod1* were partially dissected 24 h after pollination and immediately fixed with 3% glutaraldehyde in 100 mM sodium phosphate buffer, pH 7.0. After an initial fixation for 1 h at room temperature under a gentle vacuum, samples were kept in fresh fixative at 4 °C overnight. Pistils were then rinsed three times in sodium phosphate buffer and dehydrated through an ethanol series. After dehydration, ethanol was replaced by isoamyl acetate. Afterwards, specimens were dried with a critical point drier using liquid carbon dioxide. The dried pistils were mounted on stubs for further dissection and sputter coating with gold. Specimens were observed with a SEM (Hitachi). For stomatal aperture observation, siliques were fixed after ABA or S1P treatment.

### Molecular analysis and genetic complementation

Genomic DNA of *tod1* was extracted by CTAB method^55^. The flanking sequence of *Ds* element was isolated by thermal asymmetric interlaced PCR^21^. *TOD1* genomic DNA containing its promoter sequence was amplified with primers *TOD1*c*P*F and *TOD1*g*B*R and cloned into pCAMBIA1300 at *Pst*I and *Bam*HI sites. Total RNA was isolated from different tissues of L*er* with Trizol (Invitrogen). For first strand cDNA synthesis, 2 μg of total RNA was used for reverse transcription with M-MLV reverse transcriptase (Promega) according to the manufacturer’s instructions. RT–PCR was performed with primer pair *TOD1RT*F and *TOD1RT*R. *Arabidopsis eIF4A* was used as an internal control with primers *eIF4A*F and *eIF4A*R. PCR products were checked by 1.2% agarose gel electrophoresis. Uncropped gel images are shown in Supplementary Fig. 8. Real-time RT–PCR reactions containing SYBR Green I were performed on CFX96 real-time system (Bio-Rad). Primers *TOD1Q*F and *TOD1Q*R were used for *TOD1*. Primers *ACT2Q*F and *ACT2Q*R for *ACTIN2* were used to normalize the real-time RT–PCR data. Deletion variants 1-409 using primers *TOD1c1X*F and *TOD1c409K*R, 20-462 using primers *TOD1c20X*F and *TOD1c462K*R, and 40-462 using primers *TOD1c40X*F and *TOD1c462K*R were generated by RT–PCR, digested by *Xba*I and *Kpn*I and ligated into pCAMBIA1300-Lat52::NOSter under the control of *Lat52* promoter^56^. These constructs were introduced into plants via *Agrobacterium*-mediated transformation^57^. All primers are listed in Supplementary Table 2.

To express TOD1-eGFP under its native promoter, *TOD1* genomic DNA without the 3′-UTR was amplified using primer pair *TOD1*c*P*F and *TOD1*c*B*R, and cloned into pCAMBIA1300-eGFP with 3′-UTR of *TOD1* at the carboxy terminus of eGFP (Supplementary Table 2).

### Bioinformatic analysis

The TOD1 amino-acid sequence was used for BLASTP search through National Center for Biotechnology Information (http://www.ncbi.nlm.nih.gov/) and gene families search through PANTHER (http://www.pantherdb.org/). The phylogenetic tree of alkaline ceramidases was generated using the neighbour-joining method in MEGA4 (ref. 58). The alignment was generated by CUSTAL W with default parameters (http://npsa-pbil.ibcp.fr/cgi-bin/npsa_automat.pl?page=/NPSA/npsa_clustalw.html)^59^.

### Construction of *pTOD1::gTOD1*-*GUS* and GUS assay

*TOD1* 3′-UTR was amplified using primer pair *3UTR*F and 3*UTR*R and subcloned into pCAMBIA1300-GUS at the C terminus of *GUS*, after *Kpn*I and *Eco*RI digestion. Full-length *TOD1* genomic DNA containing its promoter sequence was amplified using primer pair *TOD1*c*P*F and *TOD1*c*B*R and cloned into the aforementioned binary vectors at the N terminus of *GUS* after *Pst*I and *Bam*HI digestion, yielding *pTOD1::gTOD1*-*GUS* (Supplementary Table 2). This construct was then transferred into Col as described above. *TOD1::gTOD1*-*GUS* transgenic plants were selected on MS medium containing 20 mg l^−1^ hygromycin. GUS staining was performed as described^60^. Mature pollen grains were spread on pollen germination medium (solid), and transferred for GUS staining after 6 h growth at 22 °C. Tissues were cleared in 20% lactic acid and 20% glycerol, and observed with a Zeiss Axioplan microscope.

### Transient transformation of tobacco pollen grains

The full-length coding sequence of *TOD1* was amplified by RT–PCR using primer pair *TOD1*c*E*F and *TOD1*c*B*R (Supplementary Table 2). The PCR product was digested with *Eco*RI and *Bam*HI, and then cloned into pBSK-*Lat52::mCherry*. The expression vector was co-transfected with green fluorescent protein (GFP)-fused endomembrane system markers^61^. Tobacco pollen grains were co-transfected by bombardment^62^.

Imaging of pollen tubes was performed using CLSM (Zeiss LSM510 META). Signals were captured using a 488-nm excitation laser for GFP, and a 543-nm excitation laser for mCherry. Images were processed with Zeiss LSM Image Browser (Release 3.2) and Photoshop.

### Expression of *TOD1* in yeast

The open reading frame of *TOD1* was amplified by PCR using primer pair *Flag*-*TOD1*F and *Flag*-*TOD1*R; *YPC1* was amplified by PCR primers *Flag-YPC1*F and *Flag-YPC1*R (Supplementary Table 2). PCR products were digested by *Kpn*I and *Eco*RI and cloned into the same restriction sites of pYes2. The expression vector pYes2-Flag-TOD1 or pYes2-Flag-YPC1 was transformed into the yeast strain Δ*ypc1*Δ*ydc1* using lithium acetate^63^. The yeast double knockout strain Δ*ypc1*Δ*ydc1* lacking endogenous yeast ceramidases YPC1p and YDC1p was provided by Cungui Mao (Medical University of South Carolina)^28^. The strain containing pYes2-Flag-TOD1 was maintained in synthetic dropout (SD) medium (SD/-Ura) with glucose (Clontech). The Flag-tagged TOD1 was induced by 2% galactose in SD/-Ura medium with raffinose.

### Microsome preparation and immunoblotting

Yeast cells were suspended in lysis buffer (25 mM Tris/HCl, pH 7.4, containing 1 mM phenylmethyl sulphonyl fluoride and cocktail) after being washed twice with ice-cold double-distilled deionized water. Cells were homogenized ten times for 40 s with 1 min chilling on ice between each homogenization with acid-washed glass beads (Sigma) using a TissueLyser (Qiagen) set at the maximum speed. The supernatant was transferred to a new Eppendorf tube after centrifugation at 700*g* for 5 min at 4 °C. Cell debris was removed by centrifugation at 1,400*g* for 5 min at 4 °C. To pellet the membrane fraction, the supernatant was centrifuged at 40,000 r.p.m. for 1 h at 4 °C in a Beckman Optimal L-100XP ultracentrifuge. The membrane fraction was rinsed gently with the lysis buffer and resuspended in lysis buffer. The protein concentration was determined by BCA Protein Assay Kit (TianGen). For immunoblotting, about 20 μg proteins was separated on a 10% SDS–polyacrylamide gel electrophoresis and transferred to a polyvinylidene difluoride membrane (Bio-Rad), followed by immunoblotting with 1:4,000 diluted mouse anti-Flag M2 antibody (F3165, Sigma-Aldrich) and 1:10,000 diluted goat anti-mouse IgG-HRP secondary antibody (CW0102, Beijing CoWin Biotech). The uncropped blot image is shown in Supplementary Fig. 8.

### Ceramidase activity assay

Ceramidase activity was assayed using fluorescent NBD-C_12_-phytoceramide (810215 P, Avanti), and NBD-C_12_-ceramide (810211 P, Avanti) as substrates. The substrate was dissolved in reaction buffers with different pHs^64^. In each reaction, 8 nM substrate in 20 μl reaction buffer was added to 20 μl of yeast microsomes (50 μg protein) in lysis buffer. CaCl_2_ was added to a final concentration of 5 mM. The reaction was performed at 37 °C for 90 min, and stopped by addition of methanol/chloroform (1:1). The mixture was vortexed for 30 s, kept at room temperature for 5 min and then centrifuged. The organic phase was collected and dried. Lipids were dissolved in 20 μl methanol/chloroform (1:2) then spotted on TLC plates (Merck). The substrate and product were separated by developing the plates in chloroform/methanol/25% ammonium hydroxide (90:30:0.5) solvent system. The NBD-fatty acid product was identified by comparison with C12-NBD-fatty acid (72963, Sigma) standard. The TLC plates were scanned using PhosphorImager (Typhoon Trio) system set at fluorescence mode. Uncropped TLC images are shown in Supplementary Fig. 8.

### Stomatal bioassays

Promotion of stomatal closure by ABA was performed with siliques. Siliques were floated in MES/KCl buffer (5 mM KCl, 10 mM MES and 50 μM CaCl_2_, pH 6.15) under illumination for 2.5 h (ref. 42), then mock control or 10 μM ABA was added, and siliques incubated for another 2 h, then fixed for SEM observation. S1P-induced promotion of stomatal closure was conducted in a similar way. The aperture ratio (width/length) was calculated.

### Turgor pressure measurement

The turgor pressure of pollen tubes was measured by incipient plasmolysis method as described^8^, with modifications. Pollen tubes after 5 h germination were pipetted into glass bottom dishes. An aliquot (60 μl) of original growth medium was collected for osmolality measurement denoting (*π*_e_)_0_. Pollen tubes were observed under CLSM. Liquid pollen germination medium with 10% sucrose and 10% mannitol was added into the growth medium until the plasma membrane retracted from the cell wall of the tube tip. An aliquot of 60 μl of solution was collected for osmolality measurement denoting *π*_i_. The osmolality was measured by Fiske Micro-Osmometer. Turgor pressure is given by *π*_i_−(*π*_e_)_0_.

### Ca^2+^ imaging of pollen tubes

To generate YC3.60-expressing *tod1* mutants, *tod1-2* was crossed with YC3.60-expressing Col^48^. The resulting F_1_ progeny was allowed to self-pollinate with limited pollen grains. The genotype of individual F_2_ plants was determined by PCR. Pollen grains from YC3.60-expressing *tod1-2* or Col were germinated on agarose medium. Imaging of growing pollen tubes was performed using CLSM with a 458-nm argon laser. Emission was measured at 470–500 for ECFP and 515-550 for Venus. Venus/ECFP ratio images were processed by Image J and Image-pro Plus.

## Author contributions

L.-Y.C., W.-J.Z. and W.-C.Y. designed the research; L.-Y.C., W.-J.Z. and D.-Q.S. performed the research; Z.-S.T. and J.L. contributed new reagents/analytic tools; L.-Y.C., D.-Q.S., W.-J.Z. and W.-C.Y. analysed the data; L.-Y.C. and W.-C.Y. wrote the paper.

## Additional information

**How to cite this article:** Chen, L.-Y. *et al*. The Arabidopsis alkaline ceramidase TOD1 is a key turgor pressure regulator in plant cells. *Nat. Commun.* 6:6030 doi: 10.1038/ncomms7030 (2015).

**Accession codes:** Illumina sequencing data associated with this study have been submitted to the National Center for Biotechnology Information Sequence Read Archive under accession code SRR1652473.

## Supplementary Material

Supplementary Information Supplementary Figures 1-8 and Supplementary Tables 1-2
