# Supplementary material for: The Arabidopsis alkaline ceramidase TOD1 is a key turgor pressure regulator in plant cells
Source: Nat Commun. 2015 Jan 16;6:6030. doi: 10.1038/ncomms7030 (PMC4309442; doi:10.1038/ncomms7030)
Supplement: Supplementary Information — Supplementary Figures 1-8 and Supplementary Tables 1-2 [file ncomms7030-s1.pdf]

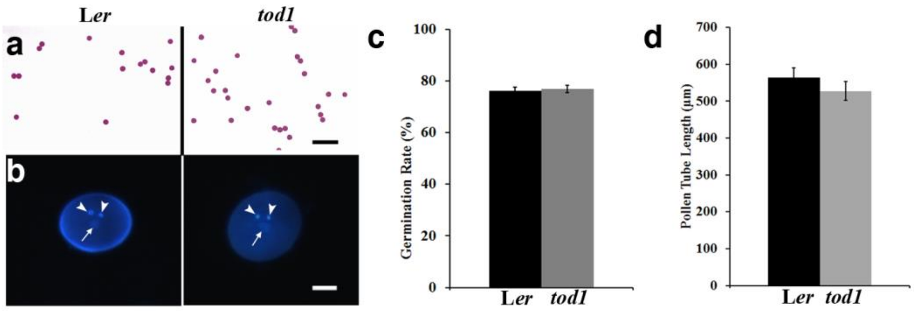

Supplemental Figure 1| Pollen development and *in vitro* pollen germination are not affected in *tod1*. (**a**) Alexander's staining of L*er* and *tod1*. Scale bar, 100 µm. (**b**) DAPI staining of L*er* and *tod1*. One vegetative nucleus (arrow) and two sperm nuclei (arrowheads) are indicated. Scale bar, 20 µm. (**c**) *in vitro* pollen germination rates of L*er* and *tod1* 5 h after germination. Data are mean ± SE (n = 5). (**d**) Pollen tube length of L*er* and *tod1* 5 h after germination. Data are mean ± SE (n = 50).

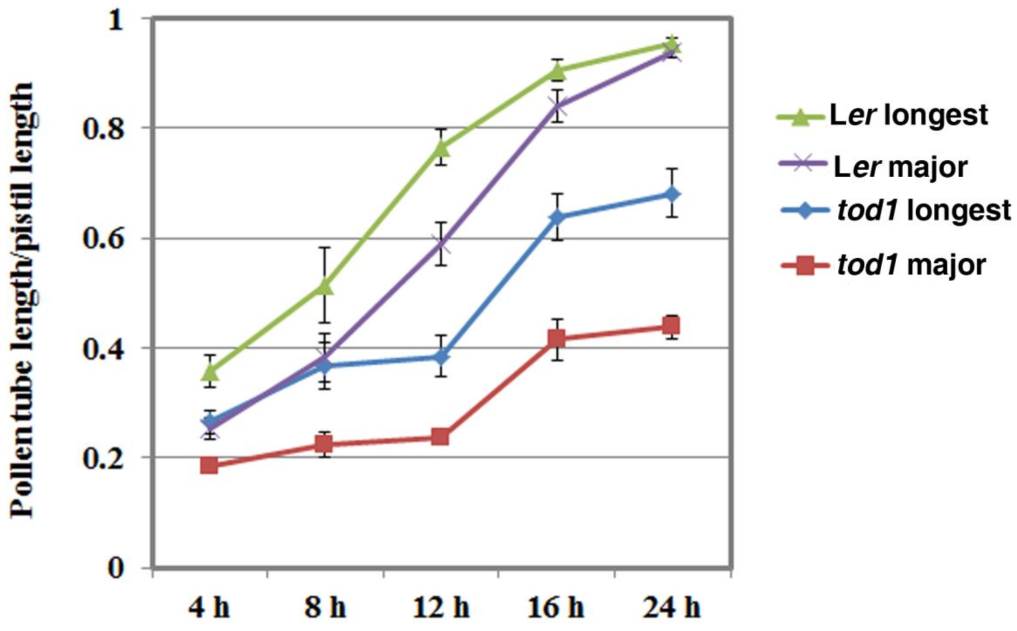

Supplemental Figure 2| *tod1* pollen tubes show growth retardation in pistil. The longest pollen tubes length or the major pollen tube length to pistil length at different times after pollination was adopted to represent pollen tube phenotype in pistil. Data are mean ± SE ($n \geq 9$).

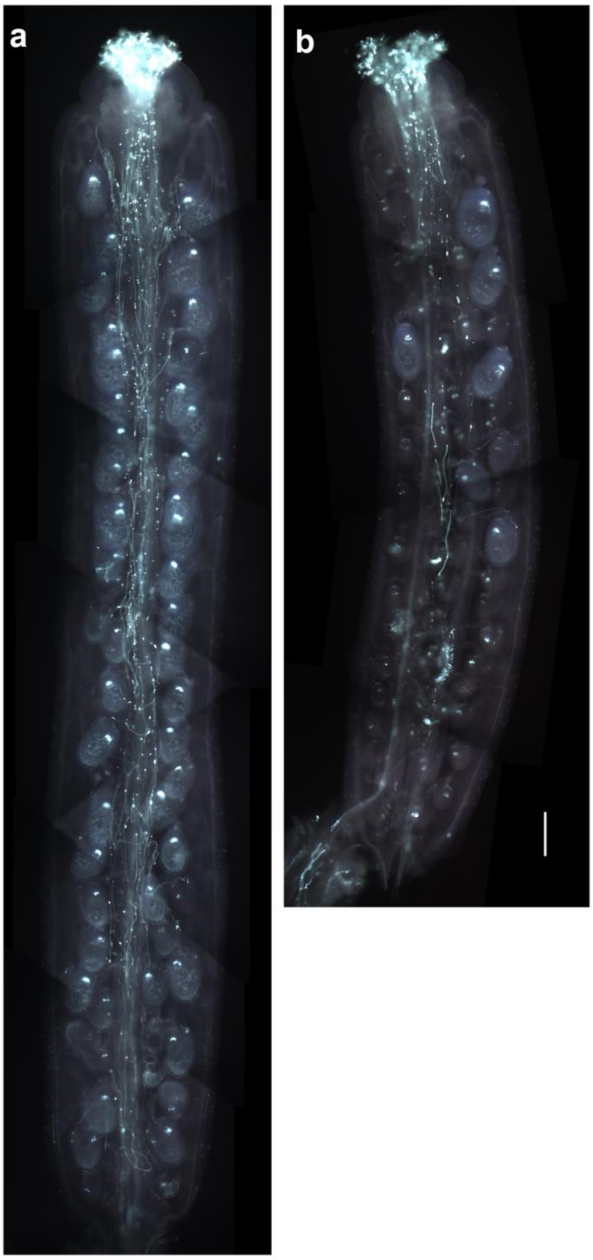

Supplemental Figure 3| The majority of *tod1* pollen tubes were still in the top half of the pistil 48 h after pollination. L*er* pollen tubes (**a**) and *tod1* pollen tubes (**b**) in L*er* pistils stained with aniline blue 48 h after pollination. Scale bar, 200 µm.

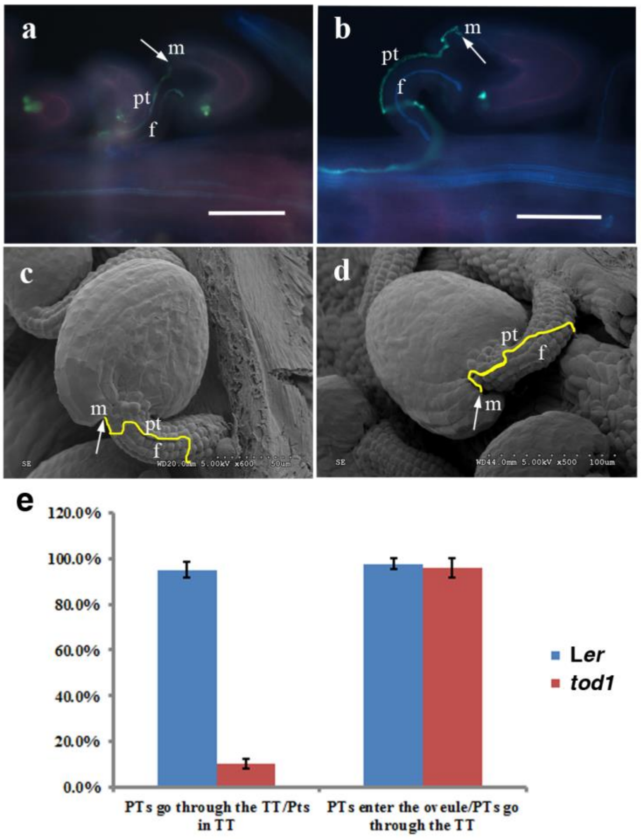

Supplemental Figure 4| Pollen tube guidance at gametophytic phase is not affected in *tod1*. Aniline blue stained L*er* (**a**) and *tod1* (**b**) pollen tubes grow along the funiculus and then enter the micropyle. Arrows indicate pollen tubes enter the micropyle. Scale bars, 100 µm. Scanning electron micrographs show that L*er* (**c**) and *tod1* (**d**) pollen tubes grow along the funiculus and then enter the micropyle. Pollen tubes are colored in yellow. m, micropyle; f, funiculus; pt, pollen tube. (**e**) Percentage of pollen tubes (PTs) that could go through the transmitting tract (TT) and percentage of ovule-invading pollen tubes among the septum-penetrating pollen tubes. Limited pollen grains (~20) were pollinated on L*er* pistil, and pistils were stained with aniline blue 24 h after pollination. Data are mean $\pm$ SE (n > 10).

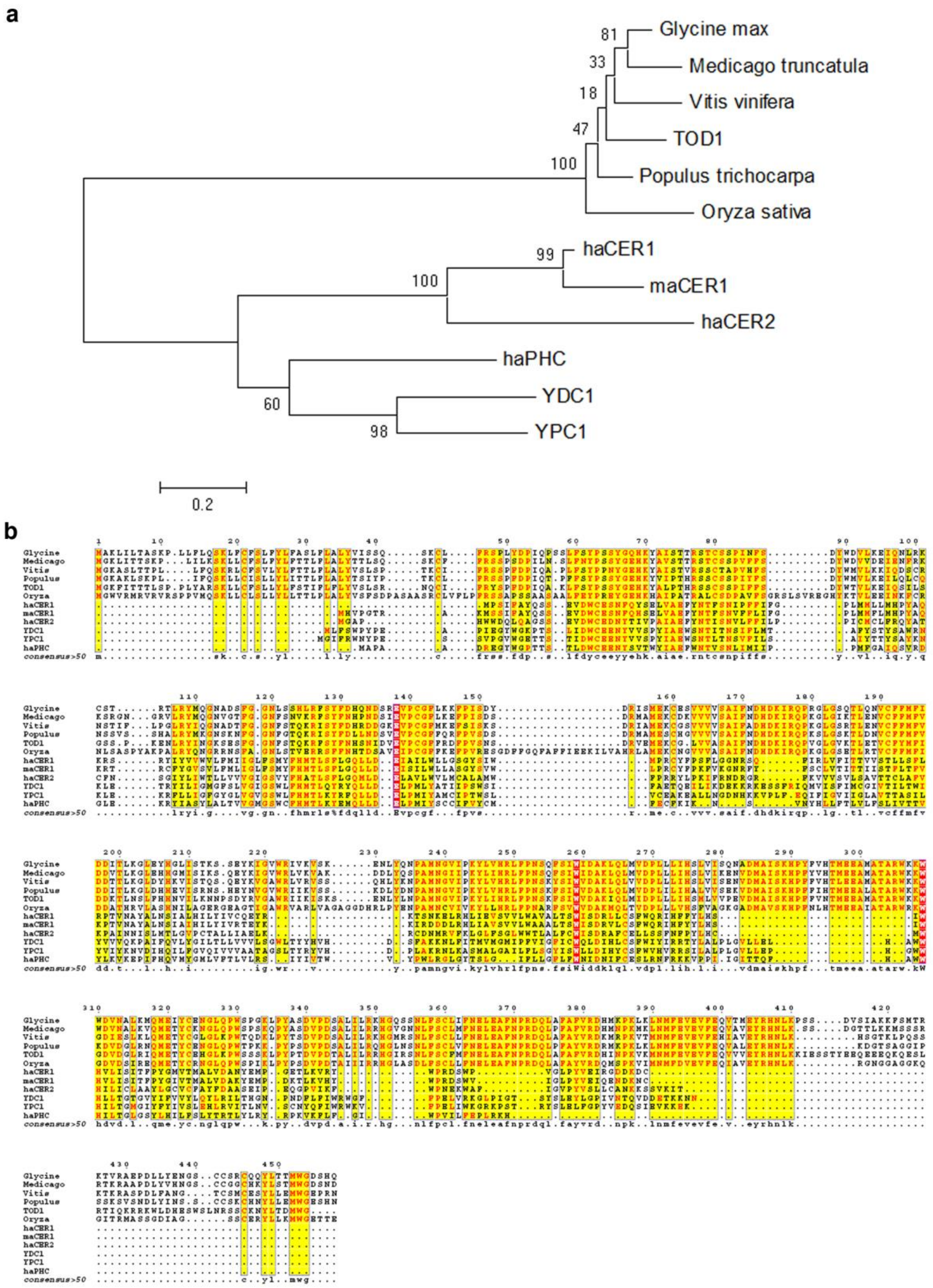

Supplemental Figure 5| Phylogenetic analysis of alkaline ceramidase family. (**a**) Phylogenetic tree of ceramidase proteins. Full length amino acids were aligned and used to generate phylogenetic tree using MEGA4. The scale bar represents 0.2 substitutions per site. (**b**) Alignment of ceramidase proteins. Species names and GenBank/EMBL accession numbers are as follows: *Arabidopsis thaliana* (TOD1, At5G46220); *Saccharomyces cerevisiae* (YDC1, AAG22594); *Saccharomyces cerevisiae* (YPC1, AF191745); *Homo sapiens* (haPHC, AAK71923); *Homo sapiens* (haCER1, NP_597999); *Homo sapiens* (haCER2, NP_001010887); *Mus musculus* (maCER1, NP_783858); *Vitis vinifera* (XP_002269609); *Glycine max*

(XP_003524117); *Medicago truncatula* (AES64180); *Populus trichocarpa* (EEF03106); *Oryza sativa* (BAF04281).

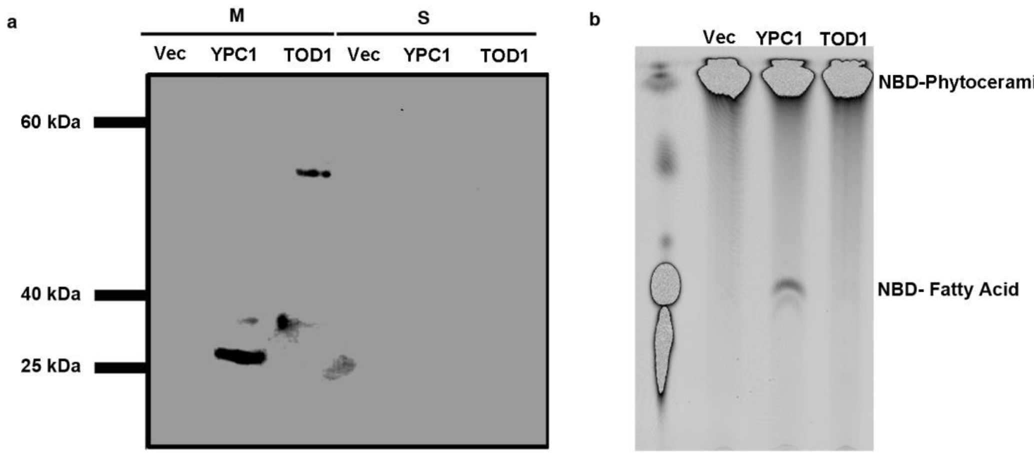

Supplemental Figure 6| TOD1 is detected in total microsomes and it is not an alkaline phytoceramidase. (**a**) Total microsomes or supernatant from Δ*ypc1*Δ*ydc1* containing empty vector pYes2 (Vec), pYes2-Flag-YPC1 (YPC1) or pYes2-Flag-TOD1 (TOD1) were tested by the Westen blot. M: total microsomes; S: supernatant. (**b**) TOD1 shows no enzyme activity towards phytoceramide at pH 9.4. Total microsomes from Δ*ypc1*Δ*ydc1* containing empty vector pYes2 (Vec), pYes2-Flag-YPC1 (YPC1) or pYes2-Flag-TOD1 (TOD1) was incubated with the substrate NBD-phytoceramide. The first lane is the NBD-fatty acid standard.

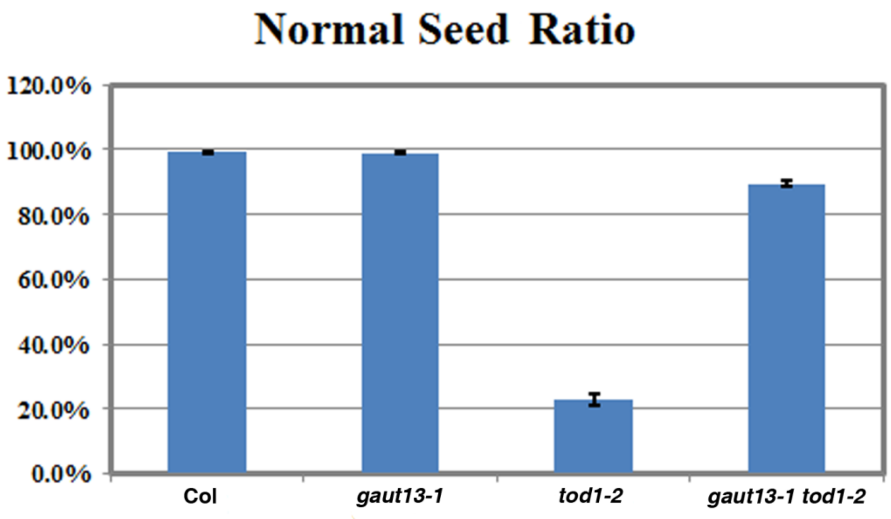

Supplemental Figure 7| Seeds ratio of Col, *gaut13-1*, *tod1-2* and *tod1-2 gaut13-1*. The seed ratio of *tod1-2 gaut13-1* double mutant plants recovers from 23% of *tod1-2* single mutant to 89%. Data are mean ± SE (n ≥ 20).

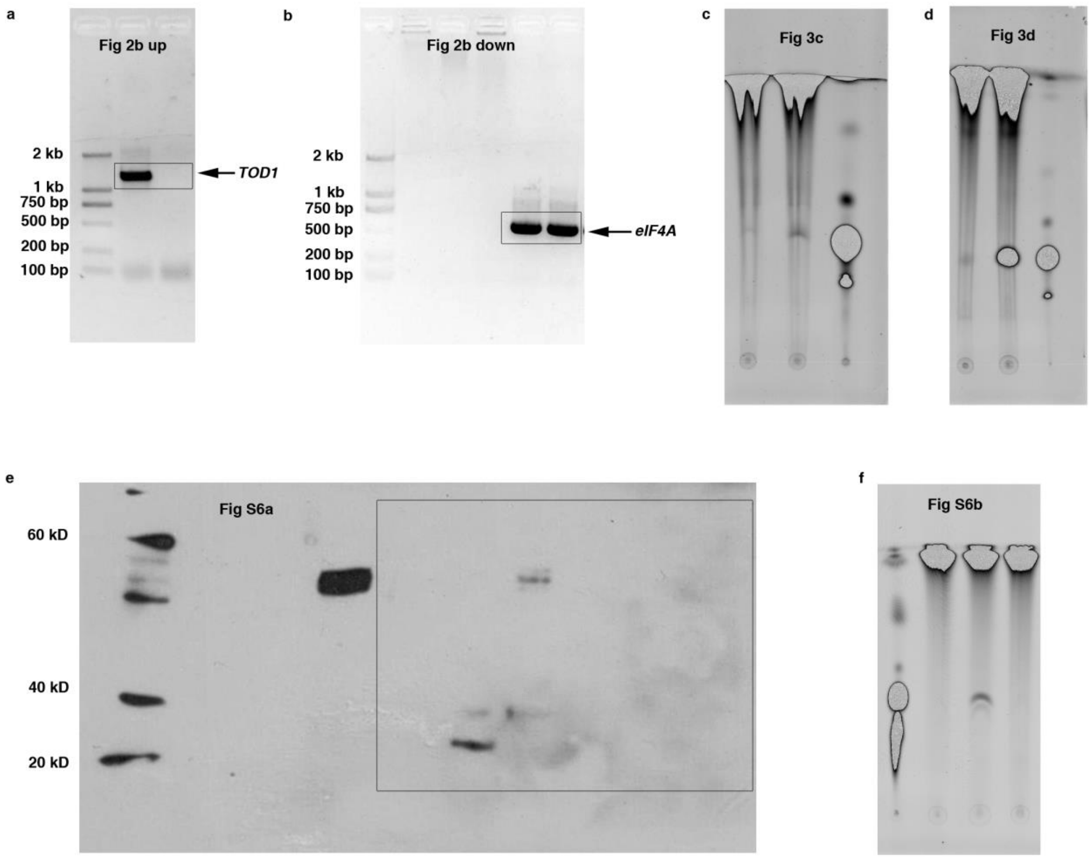

Supplemental Figure 8| Uncropped images.

Supplemental Table 1 Complementation of *tod1* using *TOD1* genomic DNA

Type	Silique Length (cm)	Normal Seed Ratio (%)
**T1**	0.97 ± 0.02	77.3 ± 2.4
**L*er***	1.23 ± 0.01	99.9 ± 0.1
***tod1*$^{(-/-)}$**	0.65 ± 0.01	18.1 ± 1.2
***tod1*$^{(+/-)}$**	1.14 ± 0.01	87.5 ± 1.8

T1, T1 generation of transgenic lines for complementation. Data are mean ± SE (n ≥ 33).

Supplemental Table 2 List of primers used in this study

**Primer name**	**Primer sequence (from 5' to 3')**
*TOD1cP*F	AACTGCAGGACATATACCGCAATGATTAGATG
*TOD1*g*B*R	CGGGATCCGTATTTCTGCATCTTTTCCATTAGAG
*TOD1RT*F	GGGAAAATTCATCACAACCAG
*TOD1RT*R	CTTGTTCCTCATACGTAGAGG
eIF4A-F	ATGGCAGGATCCGCACCGGAAGG
eIF4A-R	GCATGTCAAAAACACGACCGGGAGTTCC
*TOD1Q*F	ACACGGAATCAGAAGCAACC
*TOD1Q*R	GATCCCTCACAAACGCAAAC
*ACT2Q*F	CCAACAGAGAGAAGATGACT
*ACT2Q*R	ATGTCTCTTACAATTTCCCG
*TOD1c1X*F	GCTCTAGAATGGGAAAATTCATCACAAC
*TOD1c409K*R	GGGGTACCTTAGTGTCTGTATTCAACAACTAC
*TOD1c20X*F	GCTCTAGAATGCTCTGTTTTTCACTTCTCTAC
*TOD1c462K*R	GGGGTACCTTAGCCCCACATATCCGTG
*TOD1c40X*F	GCTCTAGAATGTCAAGAAACCAATGCATCTTTC
*TOD1cB*R	CGGGATCCGCCCCACATATCCGTGAGA
*3UTR*F	GGGGTACCCGGATATGTGGGGCTAAAACTC
*3UTR*R	GGAATTCGTATTTCTGCATCTTTTCCATTAGAG
*TOD1cE*F	GGAATTCATGGGAAAATTCATCACAACCAC
*TOD1cB*R	CGGGATCCGCCCCACATATCCGTGAGA
*Flag-TOD1*F	CGGGGTACCATGGACTACAAGGACGACGATGATAAGGGAAAATTCATCACAACCAGAC
*Flag-TOD1*R	CGGGAATTCTTAGCCCCACATATCCGTGAGATAATTCTTAC
*Flag-YPC1*F	CGGGGTACCATGGACTACAAGGACGACGATGATAAGGGAATATTTCGTTGGAACTATC
*Flag-YPC1*R	CGGGAATTCTTACTTCTCCTTTTTAACTTCAATTGATTGATC
